# Enhancing the Photocatalytic Performance of Antibiotics Using a Z-Scheme Heterojunction of 0D ZnIn_2_S_4_ Quantum Dots and 3D Hierarchical Inverse Opal TiO_2_

**DOI:** 10.3390/molecules28207174

**Published:** 2023-10-19

**Authors:** Li-Bang Zhu, Shou-Nian Ding

**Affiliations:** School of Chemistry and Chemical Engineering, Southeast University, Nanjing 211189, China; libang-zhu@seu.edu.cn

**Keywords:** photo-degradation, quantum dots, inverse opal structure, slow photons effect, Z-scheme

## Abstract

Limited light absorption and rapid photo-generated carriers’ recombination pose significant challenges to the practical applications of photocatalysts. In this study, we employed an efficient approach by combining the slow-photon effect with Z-scheme charge transfer to enhance the photo-degradation performance of antibiotics. Specifically, we incorporated 0D ZnIn_2_S_4_ quantum dots (QDs) into a 3D hierarchical inverse opal (IO) TiO_2_ structure through a facile one-step process. This combination enhanced the visible light absorption and provided abundant active surfaces for efficient photo-degradation. Moreover, the ZnIn_2_S_4_ QDs formed an artificial Z-scheme system with IO-TiO_2_, facilitating the separation and migration of charge carriers. To achieve a better band alignment with IO-TiO_2_, we doped Ag into the ZnIn_2_S_4_ QDs (Ag: ZIS QDs) to adjust their energy levels. Through an investigation of the different Ag contents in the ZnIn_2_S_4_ QDs, we found that the optimal photo-degradation performance was achieved with Ag (2.0): ZIS QDs/IO-TiO_2_, exhibiting degradation rates 19.5 and 14.8 times higher than those of ZnIn_2_S_4_ QDs and IO-TiO_2_, respectively. This study provides significant insights for elevating the photocatalytic capabilities of IO-TiO_2_ and broadening its prospective applications.

## 1. Introduction

Widely applied in human disease treatment, animal husbandry, poultry farming, and aquaculture, antibiotics have been one of the most important drugs in the world [1,2,3,4]. However, their overuse and misuse have led to the emergence of antibiotic-resistant bacteria, which is a major global health threat [5,6,7]. Antibiotics have also been found in the environment, particularly in water bodies, which can have harmful effects on the ecological balance and potentially pose a risk to human health [8,9,10]. Furthermore, antibiotics are difficult to self-degrade in natural ecosystems, making it essential to study methods for breaking them down into small molecules [11].

Over the past decades, various methods have been developed to address this issue, including biodegradation [12,13,14,15], ozonation [16,17], and photocatalysis [18,19,20,21], etc. Among the diverse methods, photocatalysis has received significant attention as a highly promising approach for treating antibiotic contaminants. This is primarily due to its cost-effectiveness and environmentally friendly nature, as it utilizes ambient conditions and sunlight to degrade antibiotics [22]. Semiconductor materials have demonstrated exceptional performances as photocatalytic materials. As early as 1972, the Japanese scholars Fujishima et al. first reported TiO_2_ as a photocatalytic electrode for splitting water [23]. Since then, TiO_2_ has been widely used in photocatalysis due to its unique properties (e.g., chemical stability, non-toxic, strong tenability, environmentally friendly, and economically viable) [24,25]. However, there are still challenges in optimizing the photocatalytic efficiency of TiO_2_, such as improving its visible light response and reducing the recombination of electron–hole pairs [26].

The inverse opal structure is a well-defined three-dimensional periodic arrangement of pores that are interconnected by thin walls, with the size of the pores and thickness of the walls being adjustable through the fabrication process. This unique structure offers several advantages for photocatalysis [27,28]. Firstly, the high surface area of the inverse opal provides numerous active sites for photocatalytic reactions, allowing for an enhanced catalytic efficiency [29]. Secondly, the periodic arrangement of the pores and walls creates a photonic bandgap, leading to improved light absorption and scattering within the structure [30,31,32]. This enhanced light–matter interaction can further boost the photocatalytic performance of the material [33]. Overall, preparing TiO_2_ in an inverse opal structure (IO-TiO_2_) could effectively improve its photocatalytic performance. While IO-TiO_2_ has indeed shown an improved photocatalytic performance compared to bulk TiO_2_, further enhancements in its catalytic performance are still necessary. The key to addressing this issue is promoting the charge flow in the photocatalyst [34]. A promising approach is to construct a heterojunction by combining IO-TiO_2_ with another semiconductor. An artificial heterojunction can generate a built-in electric field (BIEF) due to the mismatch in Fermi levels between the two materials. The presence of a BIEF can significantly facilitate the separation and collection of photo-generated carriers, ultimately improving the photocatalytic performance [35].

ZnIn_2_S_4_, a ternary semiconductor, is known for its narrow bandgap (~2.02–2.59 eV), suitable redox potentials, and good chemical stability, and it can absorb light with wavelengths up to 614 nm [36]. As a heavy-metal-free semiconductor, it is a promising material for constructing a TiO_2_-based heterojunction, extending the light absorption range of TiO_2_ and enhancing its photocatalytic efficiency by creating a BIEF. Various structural types of TiO_2_-based ZnIn_2_S_4_ photocatalysts have been developed, including nanofibers [37], nanosheets, nanoflowers [38], and hollow nanospheres [39]. However, the chemical synthesis processes used to create TiO_2_-based ZnIn_2_S_4_ photocatalysts often struggle to achieve the perfect contact between different materials (1D/2D, 2D/2D, and 2D/3D) at the nano-level in practical situations [40]. This imperfect contact can limit the efficiency and effectiveness of the heterojunction. Therefore, it is crucial to develop a method for constructing a heterojunction with perfect contact, ensuring the optimal interaction between the materials and maximizing the photocatalytic performance. Quantum dots (QDs) possess distinct properties that deviate significantly from their bulk materials [41]. QDs are confined in all three spatial dimensions, which leads to a quantum confinement effect and enables the tunability of band gaps [42]. This effect is most pronounced when the size is smaller than the excitonic Bohr radius of the particular QDs, and becomes less pronounced beyond this length scale. Different from 1D, 2D, or 3D materials, 0D QDs would be uniform and tightly attached to the inverse opal skeleton [43].

Herein, building upon our previous work about CdS quantum dots incorporated into hierarchical IO-TiO_2_ [44], we further investigated a series of heavy-metal-free 0D group II–III–VI (II–Zn/III–In/VI–S) ZnIn_2_S_4_ quantum dots (QDs). These ZnIn_2_S_4_ QDs were introduced to construct perfect contact 0D/3D ZnIn_2_S_4_ QDs/IO-TiO_2_ (denoted as ZIS QDs/IO-TiO_2_) based on a Z-scheme mechanism. Guided by the reported bandgap-tuning works, group I element Ag was doped into ZIS QDs to tune up the QDs’ band gap. Inspired by previous work, Ag-ZIS QDs/IO-TiO_2_ was synthesized via a simple one-step process and demonstrated an excellent photocatalytic degradation of antibiotics under visible light illumination [45]. Various physicochemical characterizations were conducted to investigate the advantages of coupling the slow photon effect and Z-scheme charge transfer. The superior catalytic activity observed in the Ag-ZIS QDs/IO-TiO_2_ heterojunction can be attributed to the efficient separation of the photo carriers, as supported by the corresponding characterization results. This coupling strategy opens up avenues for the development of efficient solar energy conversion devices, environmental remediation technologies, and other advanced applications that require improved light harvesting and charge transfer capabilities, making it a promising candidate for photocatalytic degradation applications.

## 2. Results and Discussion

### 2.1. Fabrication Procedure of As-Prepared IO-TiO_2_

**Scheme 1:**
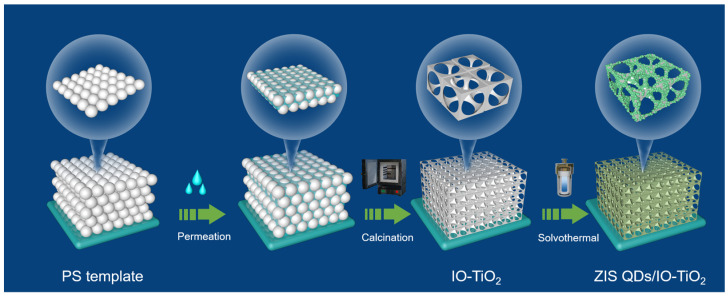
The fabrication procedure of as-prepared IO-TiO_2_.

Scheme 1 illustrates the comprehensive process of fabricating Ag-ZIS QDs/IO-TiO_2_. As shown, the fabrication entails a multi-step process, commencing with the formation of a self-assembled polystyrene (PS) sphere template. Subsequently, the voids amidst the PS spheres are infused with the TiO_2_ precursor solution. Upon complete void filling, the template undergoes high-temperature calcination, leading to the removal of PS spheres and the emergence of an inverse opal structure. Following a simple one-step hydrothermal synthesis, Ag-ZIS QDs are immobilized onto the IO-TiO_2_ surface.

### 2.2. Characterizations of As-Prepared IO-TiO_2_

**Figure 1:**
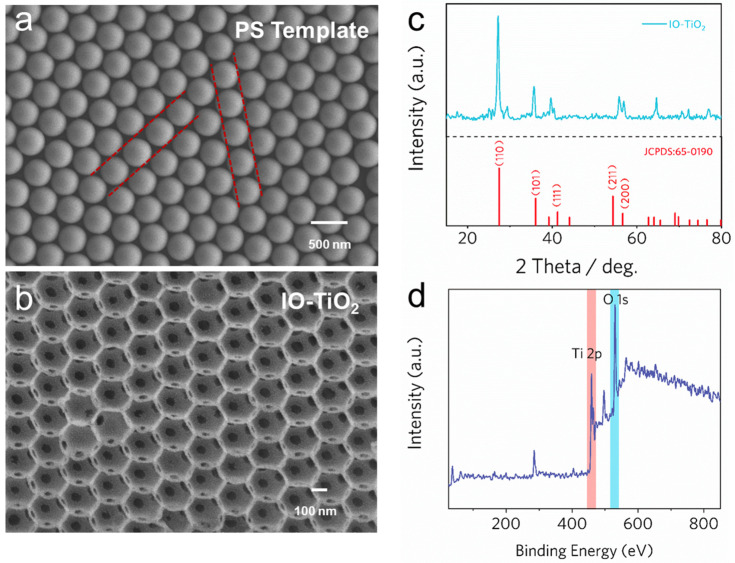
(**a**) SEM image of PS microspheres template, (**b**) prepared IO-TiO_2_, and (**c**) X-ray diffraction (XRD) pattern of IO-TiO_2_. (**d**) XPS spectra of the IO-TiO_2_.

SEM is employed to scrutinize the morphologies of the PS opal crystal templates and IO-TiO_2_, as depicted in Figure 1a,b. As shown, the PS spheres exhibit an average diameter of approximately 300 nm. Post-centrifugation, the PS spheres undergo self-assembly to establish a face-centered (fcc) periodic arrangement (Figure 1a). Following sintering, the templates are extracted, resulting in an IO-TiO_2_ framework with an average size of around 200 nm, roughly 30% smaller than that of the unadorned PS spheres (Figure 1b). This size reduction is an inevitable consequence of calcination, potentially leading to isotropic compressive stress and the formation of crystal defects. Therefore, for attaining a stable and well-ordered IO-TiO_2_ framework, it is imperative to judiciously control the heating rate during sintering, ensuring it is not excessively rapid. An X-ray powder diffraction (XRD) analysis was conducted to elucidate the phase structures of the examined samples. The obtained diffraction patterns, as depicted in Figure 1c, distinctly unveil characteristic peaks corresponding to anatase TiO_2_ (JCPDS No. 84-1286) for IO-TiO_2_. The XRD analysis unmistakably confirmed the presence of TiO_2_. The XPS spectra confirmed the presence of Ti 2p and O 1s signals (Figure 1d). Specifically, the binding energies of Ti 2p_3/2_ and 2p_1/2_, corresponding to Ti^4+^, were measured at 458.1 eV and 463.6 eV, respectively (Figure S1). The O 1s XPS spectrum exhibited three distinct peaks, reflecting the presence of three distinct oxygen chemical states within the as-prepared sample. All the characterizations confirmed the as-prepared samples’ unaltered compositions without any chemical changes.

### 2.3. Proof of the Slow-Photon Effect

The photonic bandgap (PBG) wavelength (λ) in the inverse opal (IO) structure can be determined using Bragg’s law Equation (1):

(1) $$\lambda =2\sqrt{\frac{2}{3}}D\sqrt{n_{{TiO}_{2}}^{2}f+n_{void}^{2}\left(1-f\right)-sin\theta }$$

**Figure 2:**
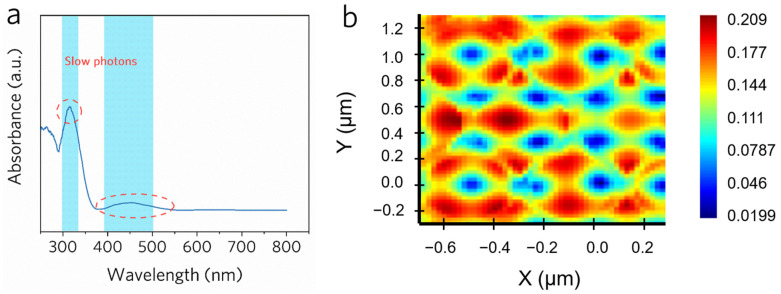
(**a**) UV–Vis DRS spectrum of prepared IO-TiO_2_. (**b**) Simulated photon transfer in IO-TiO_2_.

In the equation, *D* represents the aperture of IO-TiO_2_, while n signifies the refractive index, and *f* corresponds to the volume percentage of TiO_2_, typically assumed as 0.26. The angle of incidence of light is denoted by *θ*. At an angle of incidence of 0 degrees, the wavelength (λ) is solely governed by the pore size (*D*). UV-vis diffuse reflectance spectroscopy (DRS) elucidated multiple Bragg reflection peak positions of the synthesized IO-TiO_2_, centered at 477 and 322 nm (Figure 2a). To corroborate the presence of the photonic band gap (PBG), theoretical simulations were conducted using Optiwave OptiFDTD 7 (Figure 2b). Leveraging the finite-difference time-domain (FDTD) method, we computed the electromagnetic field distribution by solving Maxwell’s equations. The simulated distribution of the electromagnetic field intensity is depicted in Figure 2b, clearly indicating that the intensified electromagnetic fields were localized near the interfaces of IO-TiO_2_ [46].

### 2.4. Characterizations of As-Prepared Ag-ZIS QDs/IO-TiO_2_

**Figure 3:**
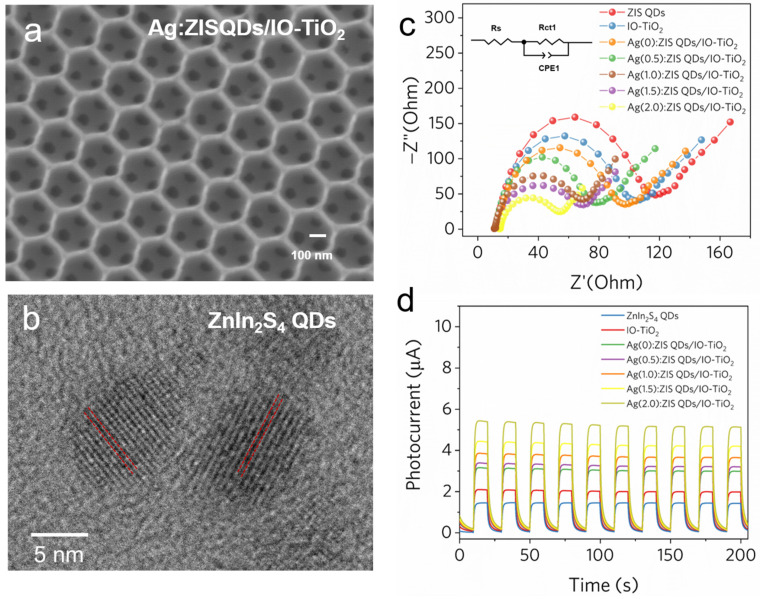
SEM image of (**a**) Ag: ZIS QDs/IO-TiO_2_, and (**b**) HRTEM image of Ag-ZIS QDs. (**c**) Electrochemical impedance spectroscopy (EIS) and (**d**) photocurrent of Ag-ZIS QDs and various content Ag: ZIS QDs/IO-TiO_2_.

Upon the integration of Ag-ZIS QDs with IO-TiO_2_, the initially smooth surface of IO-TiO_2_ underwent a transformation into a rough texture, as observed in Figure 3a. This transformation was indicative of the inverse opal structure’s framework providing an ample number of spatial sites for the immobilization of Ag-ZIS QDs. The UV–Vis spectra of IO-TiO_2_, loading various Ag contents of ZnIn_2_S_4_ QDs (Figure S2), directly prove the expansion of the light absorption range. Notably, the minute size of the Ag-ZIS QDs poses challenges for observation via SEM. To address this, TEM images were employed to scrutinize the presence and distribution of the Ag-ZIS QDs within the composite. The high-resolution transmission electron microscopy (HRTEM) image of the Ag-ZIS QDs, presented in Figure 3b, distinctly illustrates their uniform size range of 5–10 nm, revealing an absence of agglomeration. Furthermore, this image showcases a clear lattice fringe characterized by a spacing of 0.325 nm, aligning precisely with the (102) lattice plane of the hexagonal ZnIn_2_S_4_ phase. Digital photographs of all the QD samples are shown in Figure S3. In order to verify the electrochemical performance of the composite material, electrochemical impedance spectroscopy (EIS) was carried out. Figure 3c illustrates the results obtained for the unmodified Ag-ZIS QDs, IO-TiO_2_, and their respective composites. Notably, the composite Ag (2.0): ZIS QDs/IO-TiO_2_ displayed the smallest semicircle, while the semicircle associated with Ag-ZIS QDs was the largest in comparison. The reduction in the semicircle’s size for Ag (2.0): ZIS QDs/IO-TiO_2_, in contrast to that of the pristine IO-TiO_2_ and Ag-ZIS QDs, distinctly indicated the capability of the Z-scheme configuration to mitigate the interfacial charge transfer resistance. This conclusion was consistently upheld by the photocurrent response, as depicted in Figure 3d, where Ag (2.0): ZIS QDs/IO-TiO_2_ exhibits the highest photocurrent and the Ag-ZIS QDs display the lowest. The presence of Ag in ZnIn_2_S_4_ QDs can lead to an improved photocatalytic activity. This is often attributed to the creation of energy levels within the bandgap, which can act as electron traps and reduce the recombination of the photogenerated electron–hole pairs. The improved charge separation and migration in the Ag-doped ZnIn_2_S_4_ QDs resulted in more efficient photocatalysis, particularly in the degradation of organic pollutants under visible light. Evidently, all the examined materials exhibited light-responsive behavior when illuminated with a 300 W Xe lamp. The discernibly elevated photocurrent observed in Ag (2.0): ZIS QDs/IO-TiO_2_ corroborates its efficiency in charge separation and migration processes.

### 2.5. Band Structure of an Internal Electric Field

**Figure 4:**
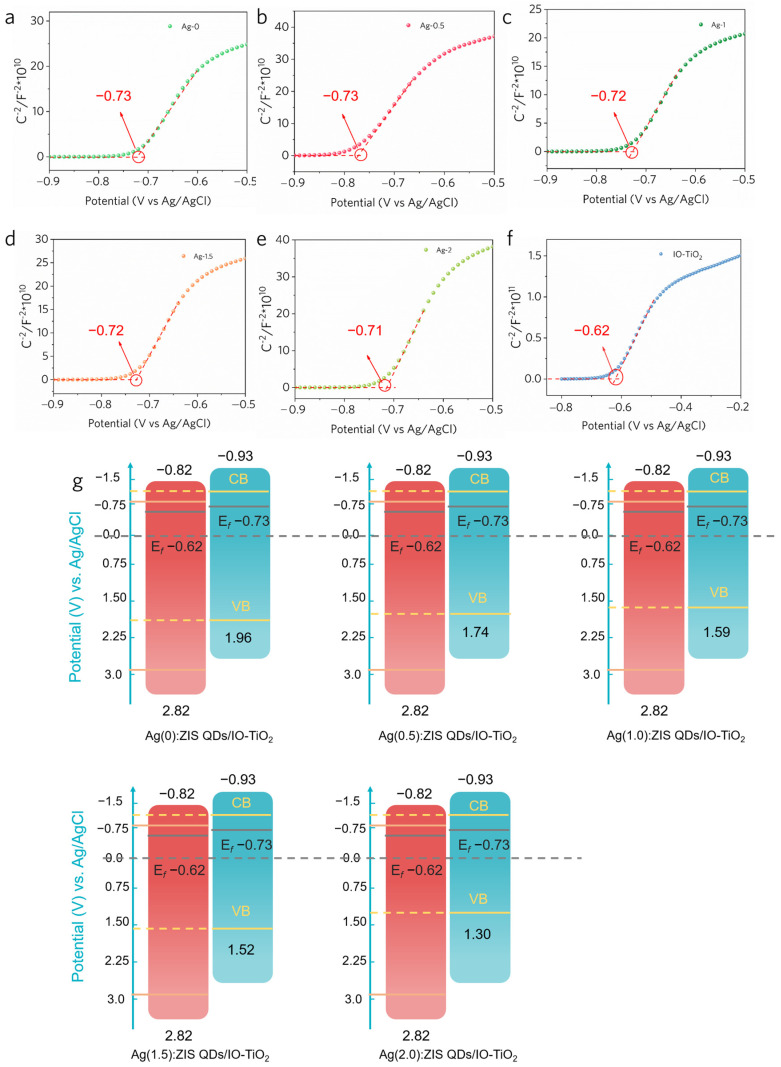
(**a**–**f**) Mott–Schottky plots of Ag (X = 0, 0.5, 1.0, 1.5, and 2.0): ZIS QDs/IO-TiO_2_, and IO-TiO_2_. (**g**) Schematic illustration band structure of IO-TiO_2_ and Ag (X = 0, 0.5, 1.0, 1.5, and 2.0): ZIS QDs.

Mott–Schottky (M-S) plots for IO-TiO_2_ and the various Ag contents of ZnIn_2_S_4_ QDs are illustrated in Figure 4a–f. Extending the linear segment of the M-S plots reveals intersections with the abscissa at −0.73, −0.73, −0.72, −0.72, and −0.71 eV vs. Ag/AgCl, corresponding to the flat band (E_fb_) of the Ag (X = 0, 0.5, 1.0, 1.5, and 2.0): ZIS QDs. Moreover, the positive slope observed in the M-S plot indicates their n-type semiconductor characteristics. Of particular note is the recognition that n-type semiconductors typically exhibit a conduction band potential (E_CB_) of approximately 0.2 eV more negative than the E_fb_. Consequently, the calculated E_CB_ values for the various ZnIn_2_S_4_ QDs and IO-TiO_2_ were −0.93, −0.93, −0.92, −0.92, −0.91, and −0.82 eV (vs. Ag/AgCl), respectively. Energy band schematic diagrams illustrating IO-TiO_2_ and the various compositions of Ag(X): ZIS QDs were constructed based on the respective bandgap (E_g_) values of all the samples presented in Figure 4g. The bandgap energy (Eg) provides information about the semiconductor’s electronic structure. Materials with smaller bandgaps are more likely to absorb visible light and are often used in photovoltaic and photocatalytic applications. The E_g_ of the various compositions of the Ag (0–2.0): ZIS QDs were determined to be 2.89, 2.67, 2.51, 2.44, and 2.21 eV through the Tauc method (See Figure S4 for detailed calculation). The fundamental principle underlying the construction of a Z-scheme heterojunction is to align the energy bands between semiconductors A and B. In this context, it is crucial for the conduction band (CB) of semiconductor A to closely match the valence band (VB) of semiconductor B [47]. Interestingly, the optimal semiconductor choice for forming a Z-scheme heterojunction with IO-TiO_2_ is Ag (2.0): ZIS QDs, which boasts an appropriately aligned VB value of ~1.30 eV, in proximity to the IO-TiO_2_ CB.

In the course of the experiment, Electron Paramagnetic Resonance (EPR) spectroscopy was employed to validate the generation of reactive oxygen species (ROS). DMPO was utilized as a spin-trapping reagent, and a subdued DMPO−·OH signal emerged in the absence of light. However, under photoexcitation, distinctive peaks corresponding to DMPO−·OH (1:2:2:1) manifested in the methanol dispersion liquid of ZnIn_2_S_4_ QDs/IO-TiO_2_ (Figure S5). These characteristic DMPO−·OH signals were observed for ZnIn_2_S_4_ QDs/IO-TiO_2_, indicating that this heterojunction maintained a strong redox ability. This was further corroborated by the results of the ESR analysis, which revealed the characteristic signals corresponding to DMPO−·O^2−^ and DMPO−·OH simultaneously, thus excluding the possibility of forming a conventional type-II mode for the ZnIn_2_S_4_ QDs/IO-TiO_2_ heterojunction. Based on these analyses, we can confidently confirm the successful construction of a direct Z-scheme heterojunction in ZnIn_2_S_4_ QDs/IO-TiO_2_. This observation firmly establishes the involvement of the charge migration route within the traditional Z-scheme transfer mechanism. 

### 2.6. Photocatalytic Activity

**Figure 5:**
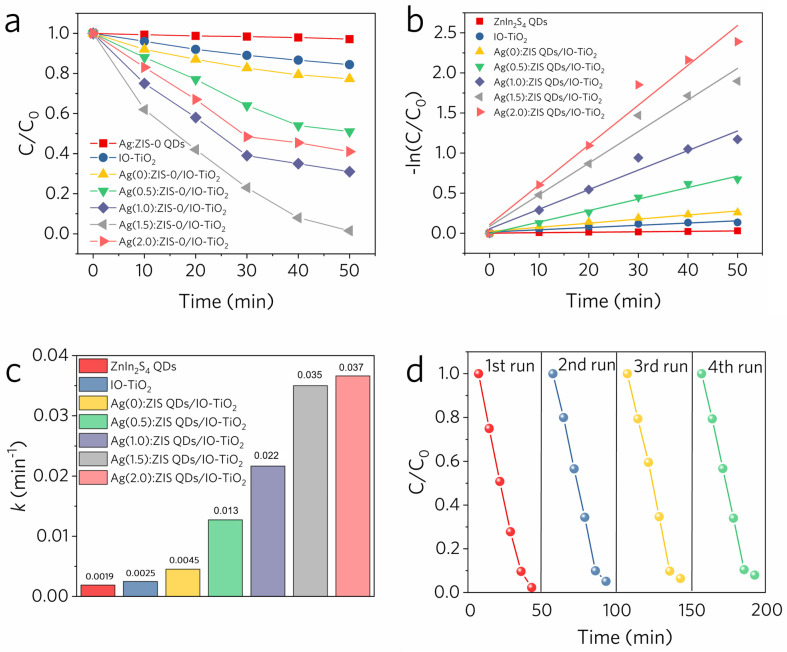
(**a**) Tetracycline degradation results of samples. (**b**) Fitting diagram of the degradation rate under visible light irradiation. (**c**) The value of rate “k”. (**d**) Cycle tests of Ag (2.0): ZIS QDs/IO-TiO_2_.

The focal point of this investigation centered on utilizing Ag-ZIS QDs/IO-TiO_2_ as a heterogeneous photocatalyst for tetracycline degradation. The outcomes, depicted in Figure 5a, reveal that the degradation of tetracycline remained minimal when exposed to single-component ZnIn_2_S_4_ QDs and IO-TiO_2_ due to their limited charge separation capacity. Additionally, the UV–Vis absorbance spectra of the composites during dark adsorption are presented in Figure S7. Remarkably, the degradation rate experienced a significant enhancement with the use of Ag-ZIS QDs/IO-TiO_2_ composites, achieving up to an 84% degradation (Ag (2.0): ZIS QDs/IO-TiO_2_, 50 min). These observations furnish compelling evidence for the substantial contribution of the Z-scheme migration mechanism to the enhanced efficiency of the Ag-ZIS QDs/IO-TiO_2_ heterojunction system. To deepen our comprehension of the photocatalytic degradation efficacy, we calculated the corresponding reaction rate constant “k” by fitting the reaction’s degradation curve using Equation (2):

(2) $$-\ln\left(C/C_{0}\right)=kt$$

Based on the fitting outcomes, the composite comprising Ag (2.0): ZIS QDs/IO-TiO_2_ demonstrated the highest degradation rate, surpassing those of the pristine ZnIn_2_S_4_ QDs (0.0019 min^−1^) and IO-TiO_2_ (0.0025 min^−1^) by 13 and 7.8 times, respectively (Figure 5c). Moreover, as catalysts, achieving both high and stable catalytic activity is paramount. Notably, even after the fourth recycling test, the prepared photocatalysts consistently maintained a remarkable level of photocatalytic activity, underscoring their excellent reusability (Figure 5d). The stability of the catalysts was also characterized using XRD, and after four recycles tests, the catalyst showed minor changes (Figure S8).

### 2.7. A Possible TC Photo-Degradation Pathway

**Scheme 2:**
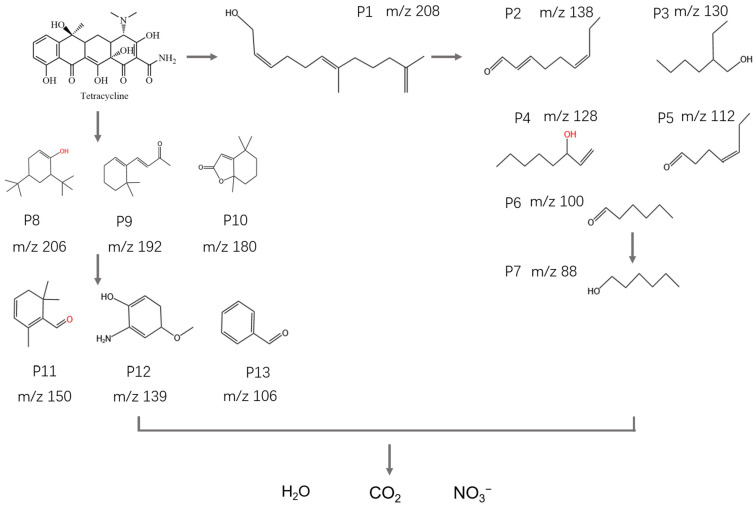
Proposed photo-degradation pathway of TC in the Ag: ZIS QDs/IO-TiO_2_ system.

To explore the photo-degradation of TC molecules into smaller compounds within the Ag: ZIS QDs/IO-TiO_2_ system following a 50 min photocatalysis reaction, we conducted LC-MS measurements. The results, depicted in Scheme 2, unveil the presence of 25 distinct intermediate products identified via a mass library analysis. It is noteworthy that no large molecule intermediate products were detected, likely attributable to the swift pace of the photocatalytic reaction. These findings align with previous TC degradation research [48], thereby permitting the formulation of a conceivable decomposition pathway for TC within the Ag: ZIS QDs/IO-TiO_2_ system. This pathway encompasses the creation of intermediate products, encompassing aldehydes (P2, P6, P11, and P13), alcohols (P1, P3, and P5), phenols (P8 and P12), and esters (P10). The genesis of these intermediates is facilitated through central carbon cracking and ring-opening reactions, entailing the removal of hydroxyl, amino, and methyl groups. Ultimately, these intermediate products within the Ag: ZIS QDs/IO-TiO_2_ system undergo further oxidation to yield H_2_O, CO_2_, and NO^3−^.

## 3. Materials and Methods

### 3.1. Reagents and Apparatus 

Silver nitrate (AgNO_3_), zinc acetate (Zn(OAc)_2_·2H_2_O), thioacetamide (TAA), indium nitrate (In(NO_3_)_3_·4.5H_2_O), and L-cysteine (HSCH_2_CH(NH_2_)CO_2_H) were purchased from Sigma-Aldrich. Sulfur powder, sodium hydroxide (NaOH), and ethanol were obtained from Sinopharm Chemical Reagent Co., Ltd. All the reagents were of analytical grade and were used as received, without any additional purification.

### 3.2. Synthesis of Ag: ZnIn_2_S_4_ Quantum Dots (Ag: ZIS QDs) 

Ag-doped ZnIn_2_S_4_ quantum dots (Ag: ZIS QDs) were synthesized using a hydrothermal method [49]. In a typical synthesis, zinc acetate dihydrate (1.7 mmol) and indium(III) acetate (3.4 mmol) were dissolved in 70 mL of distilled water to form the precursor solution. Various amounts of silver nitrate were introduced to the precursor solution to prepare the Ag (X): ZIS QDs, where X represents the molar ratios of the doped silver. Subsequently, an aqueous solution of L-cysteine (0.17 mmol) was added under vigorous stirring, and the pH of the mixture was adjusted to 8.5 using a 1.0 M NaOH solution. Excess thioacetamide (TAA) (6.5 mmol) was then rapidly added to the mixture. The resulting solution was sealed in a Teflon-lined stainless steel autoclave and heated at 110 °C for 4 h. After cooling to room temperature, the various Ag: ZIS QDs were collected through centrifugation and subsequently washed three times using a water and ethanol mixture (with a volume ratio of 30:70).

### 3.3. Synthesis of PS Microspheres

The synthesis of the PS spheres followed this procedure [50]. Initially, potassium persulfate (4 mmol) and sodium dodecyl sulfate (1.5 mmol) were dissolved in a 40 mL aqueous ethanol solution (50% *v*/*v*). The mixture was then heated to 75 °C under a nitrogen atmosphere, followed by the addition of 10 mL of styrene. Continuous stirring was maintained for 19 h, resulting in the formation of a white emulsion, creating a suspension of PS microspheres. For the fabrication of ordered PS array film templates, 500 μL of the prepared PS spheres suspension was dispersed into 25 mL of deionized water and subsequently sonicated for 30 min to ensure a uniform dispersion. Cleaned ITO-coated glass slides were then subjected to alternating ultrasonic cleaning with ethanol, acetone, and deionized water for 5 min. Following this, the slides were vertically immersed in a beaker and placed in a 75 °C oven. As the liquid evaporated, a hexagonal close-packed PS array film formed on the slides.

### 3.4. Fabrication of Ordered PS Array Film Templates

To achieve a consistent dispersion, we introduced the prepared PS spheres suspension (500 μL) into 25 mL of deionized water and subjected it to 30 min of sonication. The ITO-coated glass slides underwent a thorough cleaning process, including sequential ultrasonic treatments with ethanol, acetone, and deionized water, each lasting for 5 min.

### 3.5. Synthesis of IO-TiO_2_ Structure

The IO-TiO_2_ structure was synthesized using an infiltration method. The sacrificial templates were fabricated by filling the interstitial spaces between the PS spheres with the TiO_2_ precursor. The procedure was as follows: the TiO_2_ precursor, comprising TiBALDH, 0.1 M HCl, and ethanol (1:1:1.5 volume ratio), was stirred for 1 h at room temperature. Subsequently, 10 μL of the precursor was deposited onto the pre-treated PS template and incubated at 35 °C for 4 h. The temperature of the electrodes was then raised from room temperature to 450 °C at a ramp rate of 1.0 °C·min^−1^ and held at this temperature for 2 h, resulting in the formation of the IO-TiO_2_ structure.

### 3.6. Photocatalytic Activity Test

The photocatalytic performance of the photocatalysts was assessed by degrading TC under visible light. In each experiment, 50 mg of the Ag-ZIS QDs/IO-TiO_2_ sample was dispersed in an aqueous solution containing TC (10 mg/L). Before exposing it to light, the suspension was stirred in the dark for 1 h to establish an adsorption–desorption equilibrium between the photocatalyst and the TC. During the irradiation process, visible light was generated using a 250 W xenon lamp filtered through a UV cutoff filter (λ > 420 nm). Over the course of the photocatalytic process, 5 mL of the suspension was extracted at 30 min intervals. The concentration of TC in the solution was monitored via UV-vis spectroscopy, measuring the absorbance at a characteristic wavelength of 357 nm. The degradation efficiency was calculated using the formula −ln(*C*/*C*_0_), where *C* represents the TC concentration at each irradiation time and *C*_0_ is the initial concentration. 

### 3.7. Characterization

The structural analysis of the samples was conducted using X-ray diffraction (XRD) with X’TRA and Cu Kα (ARL Co.) (D8ADVANCE, Bruker, Salbuluken, Germany) equipment. X-ray photoelectron spectroscopy (XPS) measurements were carried out utilizing a PHI 5000 Versa Probe (UlVAC-PHI Co., Chigasaki, Japan). For obtaining high-resolution transmission electron microscopy (HRTEM) images of the Ag (X): ZIS QDs, a JEOL JEM-2100 transmission electron microscope (Hitachi, Tokyo, Japan) was employed.

## 4. Conclusions

In conclusion, we successfully fabricated a well-defined ZnIn_2_S_4_ QDs/IO-TiO_2_ heterojunction using a facile one-step hydrothermal process. Thorough characterizations were performed to assess its potential for solar conversion applications. The IO structure not only endowed the system with abundant active sites, but also enhanced light harvesting through the slow photon effect. The strategic confinement of 0D ZnIn_2_S_4_ QDs onto the IO structure surface further enhanced the photocatalytic capability of the nanohybrids, taking advantage of their band energy level alignment. To further optimize the photocatalytic performance, Ag was introduced as a dopant into the 0D ZnIn_2_S_4_ QDs, thereby fine-tuning their band structure. Ultrafast transient infrared absorption studies unequivocally confirmed a Z-scheme charge transfer mechanism in the optimized composition, 2% Ag: ZIS QDs/IO-TiO_2_, which adeptly harnessed the reducing power of the ZnIn_2_S_4_ QDs conduction band and the IO-TiO_2_ valence band oxidizing power. Impressively, under simulated solar irradiation, the Ag: ZIS QDs/IO-TiO_2_ heterojunction displayed degradation rates 19.5 and 14.8 times higher than those of the pristine ZnIn_2_S_4_ QDs and IO-TiO_2_, respectively. This pioneering study, amalgamating the slow photon effect and Z-scheme charge transfer, substantiated remarkable enhancements in photocatalytic performance. The discerning insights gleaned from this work could serve as a commendable blueprint for the advancement of 0D/3D catalysts across diverse applications.

## Data Availability

Not applicable.

## Supplementary Materials

The following supporting information can be downloaded at: https://www.mdpi.com/article/10.3390/molecules28207174/s1, Figure S1: X-ray photoelectron spectroscopy of O1s and Ti 2p; Figure S2: UV–Vis spectra of IO-TiO_2_ and various Ag contents of ZnIn_2_S_4_ QDs; Figure S3: Digital photograph of various Ag contents of ZnIn_2_S_4_ QDs under light (ultraviolet light); Figure S4: Tauc plots of Ag (X = 0, 0.5, 1.0, 1.5, 2.0): ZIS QDs; Figure S5: EPR spectra of DMPO-OH; Figure S6. Optimization of pH for TC photodegradation experiment; Figure S7: UV–Vis spectra of tetracycline (TC) aqueous solution in the presence of Ag (2.0): ZIS QDs/IO-TiO_2_ heterojunction under visible light irradiation; Figure S8. XRD of the catalyst before and after 4th recycle run. Table S1. Electrolyte resistance (R_S_), charge transfer resistance (R_CT_), and double-layer capacitance values (C_DL_) of Ag (X): ZIS QDs/IO-TiO_2_.

## Sample Availability

Samples of the Ag: ZIS QDs/IO-TiO_2_ are available from the authors.
