# Supplementary material for: Enhancing the Photocatalytic Performance of Antibiotics Using a Z-Scheme Heterojunction of 0D ZnIn2S4 Quantum Dots and 3D Hierarchical Inverse Opal TiO2
Source: Molecules. 2023 Oct 19;28(20):7174. doi: 10.3390/molecules28207174 (PMC10609623; doi:10.3390/molecules28207174)
Supplement: Supplementary file 1 [file molecules-28-07174-s001.zip › molecules-2615940-supplementary.pdf]

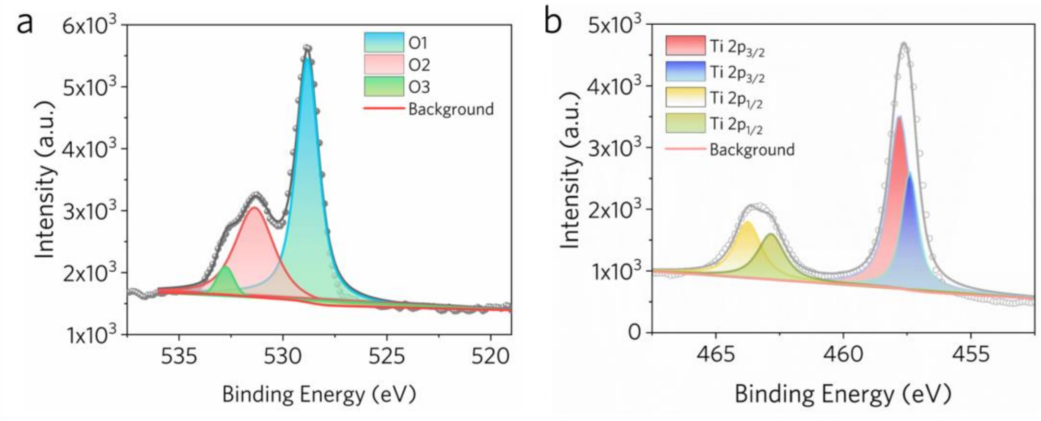

Figure S1. X-ray photoelectron spectroscopy of O1s and Ti 2p.

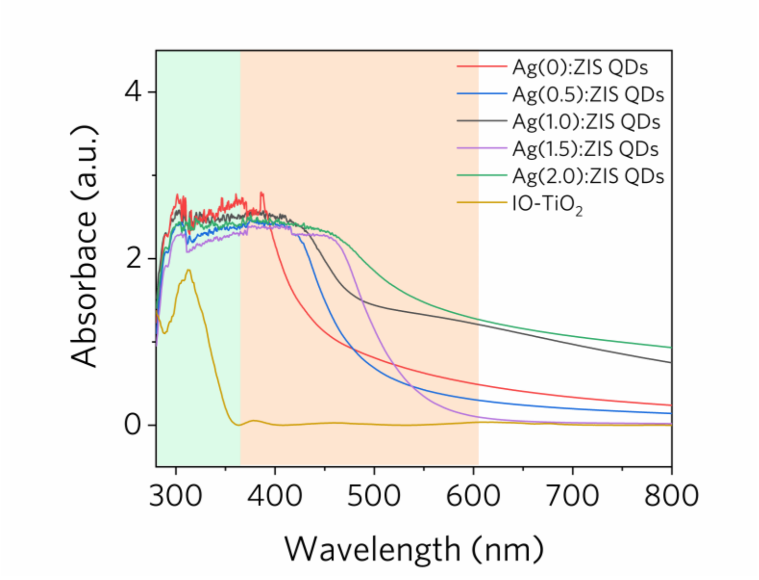

Figure S2. UV–Vis spectra of IO-TiO_2 and various Ag contents of $ZnIn_2S_4$ QDs.

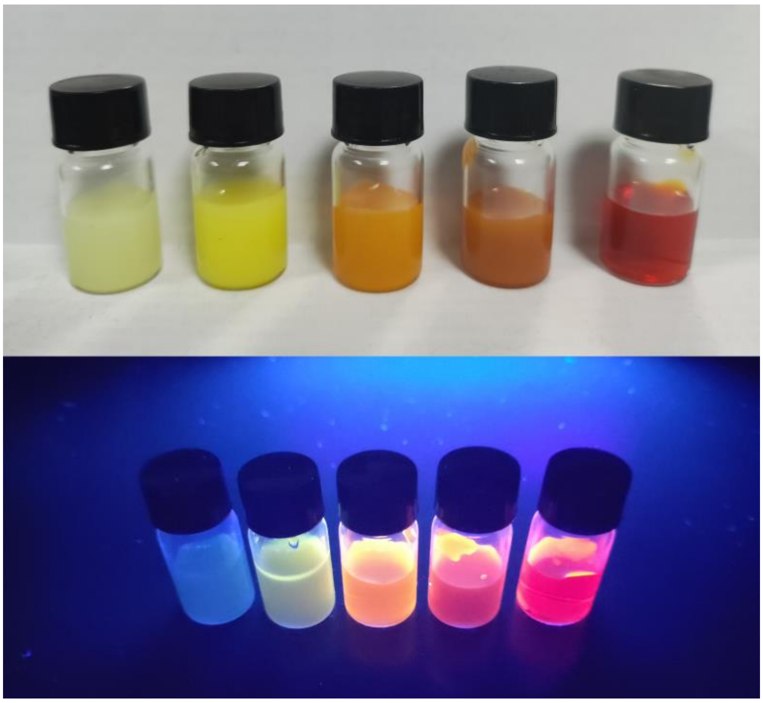

Figure S3. Digital photograph of various Ag contents of $ZnIn_2S_4$ QDs under light (ultraviolet light).

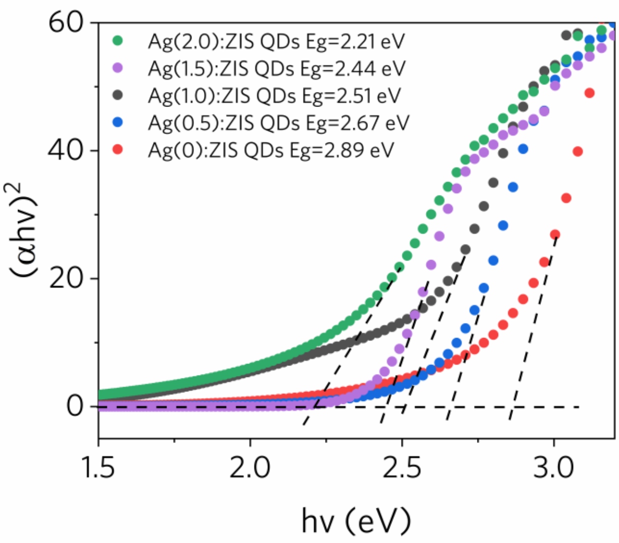

Figure S4. Tauc plots of Ag(X=0, 0.5, 1.0, 1.5, 2.0): ZIS QDs.

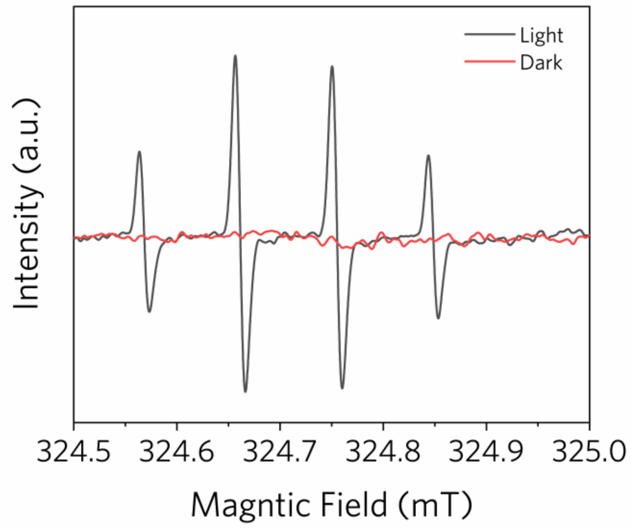

Figure S5. EPR spectra of DMPO-·OH.

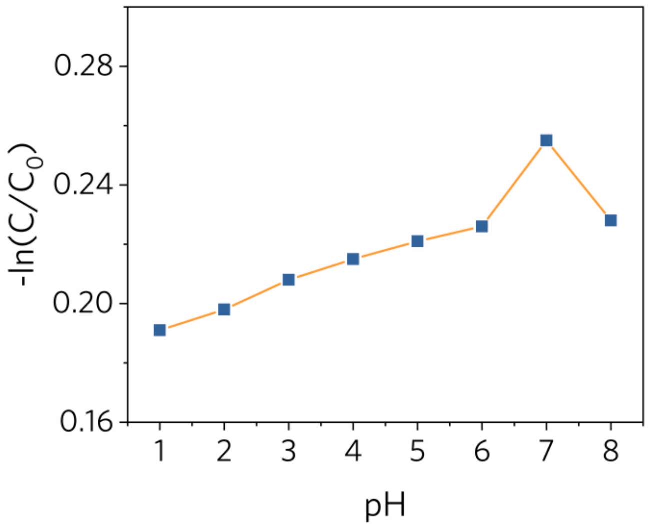

Figure S6. Optimization of pH for TC photodegradation experiment.

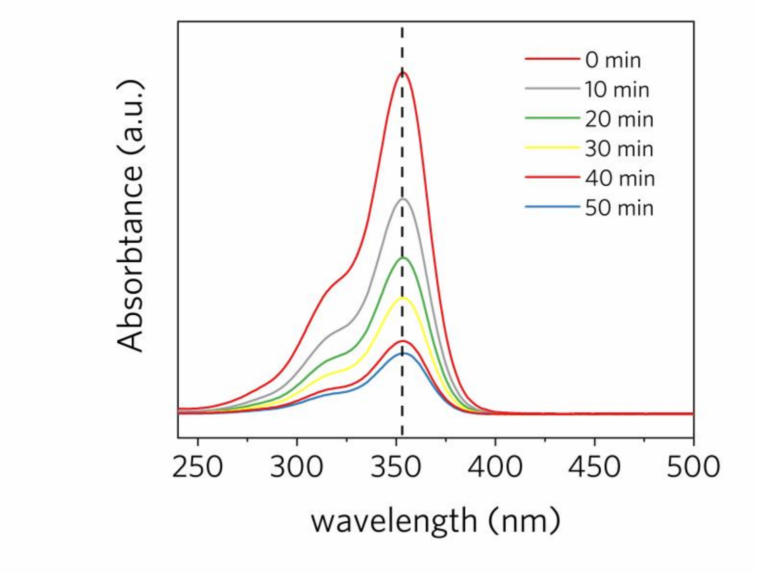

Figure S7. UV–Vis spectra of tetracycline (TC) aqueous solution in the presence of Ag(2): ZIS QDs/IO-TiO_2 heterojunction under visible light irradiation.

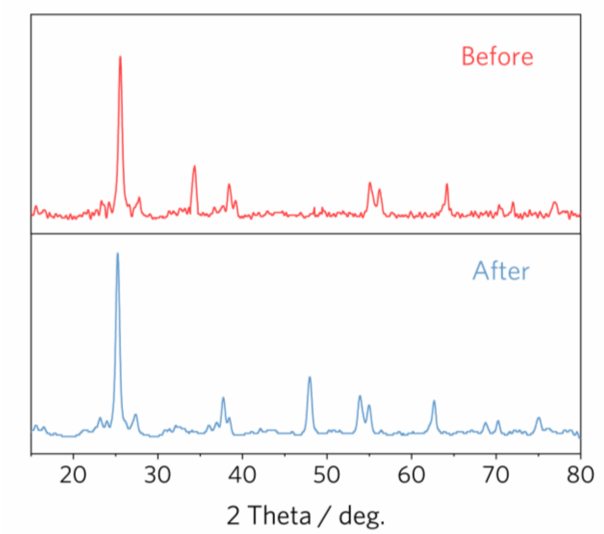

Figure S8. XRD of the catalyst before and after 4th recycle run.

Table S1. Electrolyte resistance (R_S), charge transfer resistance (R_{CT}), and double-layer capacitance values (C_{DL}) of Ag (X): ZIS QDs/IO-TiO_2.

	R_S	R_{CT}	C_{DL}
Ag (0): ZIS QDs/IO-TiO_2	9.4	107.2	1.79
Ag (0.5): ZIS QDs/IO-TiO_2	10.4	67.5	1.13
Ag (1.0): ZIS QDs/IO-TiO_2	10.6	59.6	0.99
Ag (1.5): ZIS QDs/IO-TiO_2	11.4	58.3	0.97
Ag (2.0): ZIS QDs/IO-TiO_2	15.6	7.6	0.13
